# Supplementary material for: The Effects of Antibiotic Combination Treatments on Pseudomonas aeruginosa Tolerance Evolution and Coexistence with Stenotrophomonas maltophilia
Source: Microbiol Spectr. 2022 Dec 1;10(6):e01842-22. doi: 10.1128/spectrum.01842-22 (PMC9769631; doi:10.1128/spectrum.01842-22)
Supplement: Supplemental file 1 — Supplemental material. Download spectrum.01842-22-s0001.pdf, PDF file, 0.1 MB [file spectrum.01842-22-s0001.pdf]

The effects of antibiotic combination treatments on *Pseudomonas aeruginosa* tolerance evolution and coexistence with *Stenotrophomonas maltophilia*

Jack P. Law[1] ORCID 0000-0002-2658-4601,
A. Jamie Wood[1,2] ORCID 0000-0002-6119-852X,
Ville-Petri Friman[1*] ORCID 0000-0002-1592-157X

1. Department of Biology, University of York, York, UK
2. Department of Mathematics, University of York, York, UK

* Corresponding author: ville.friman@york.ac.uk

Supplemental Materials

Supplementary Tables

Table S1: ANOVA tables for growth of evolved *P. aeruginosa* when exposed to each antibiotic in growth assays, as difference in growth with antibiotic relative to without antibiotic, $\Delta epOD_{600}^{Abx}$. Treatment term refers to the antibiotic treatment regimen, culture refers to the presence or absence of *S. maltophilia*.

Strain	Antibiotic	ANOVA term	D.F.	Sum Sq.	F value	P value
PAO1	Ciprofloxacin	Treatment	7	0.17	63.42	$<2x10^{-16}$
		Culture	1	$3.5x10^{-4}$	0.94	0.34
		Interaction	5	$2.5x10^{-4}$	0.13	0.98
		Residuals	48	0.02		
PAO1	Colistin	Treatment	7	0.06	6.07	$4.2x10^{-5}$
		Culture	1	$1.2x10^{-4}$	0.09	0.77
		Interaction	5	$4.4x10^{-3}$	0.65	0.66
		Residuals	48	0.06		
PAO1	Tobramycin	Treatment	7	0.21	16.17	$1.0x10^{-10}$
		Culture	1	$1.7x10^{-3}$	0.95	0.34
		Interaction	5	0.01	1.62	0.17
		Residuals	48	0.09		
LESB58	Ciprofloxacin	Treatment	7	0.22	8.3	$2.9x10^{-7}$
		Culture	1	$1.3x10^{-4}$	0.03	0.85
		Interaction	7	$9.1x10^{-3}$	0.35	0.93
		Residuals	66	0.24		
LESB58	Colistin	Treatment	7	0.25	10.41	$1.0x10^{-8}$
		Culture	1	$<1.0x10^{-5}$	$1.0x10^{-4}$	0.99
		Interaction	7	0.03	1.05	0.41
		Residuals	66	0.23		
LESB58	Tobramycin	Treatment	7	0.46	19.11	$1.1x10^{-13}$
		Culture	1	$8.6x10^{-4}$	0.25	0.62
		Interaction	7	0.01	0.55	0.79
		Residuals	66	0.23		

Table S2: ANOVA tables for growth of evolved *P. aeruginosa*, as difference in growth without antibiotic relative to ancestor, $\Delta epOD_{600}^{E}$. Treatment term refers to the antibiotic treatment regimen, culture refers to the presence or absence of *S. maltophilia*.

Strain	ANOVA term	D.F.	Sum Sq.	F value	P value
PAO1	Treatment	7	7.7×10^{-3}	5.06	2.3×10^{-4}
	Culture	1	1.1×10^{-6}	5.0×10^{-3}	0.94
	Interaction	5	5.7×10^{-4}	0.52	0.76
	Residuals	48	0.01		
LESB58	Treatment	7	0.02	6.25	1.24×10^{-5}
	Culture	1	2.5×10^{-5}	0.06	0.81
	Interaction	7	3.9×10^{-3}	1.39	0.23
	Residuals	66	0.03		

Table S3: Pearson Chi-Squared Test of Independence for the MIC of each antibiotic for both *P. aeruginosa* strains.

Strain	Antibiotic	X^2	D.F.	P value
PAO1	Ciprofloxacin	32.13	7	3.84×10^{-5}
	Colistin	29.00	7	1.44×10^{-4}
	Tobramycin	30.55	7	7.52×10^{-5}
LESB58	Ciprofloxacin	33.47	7	2.16×10^{-5}
	Colistin	29.89	7	9.97×10^{-5}
	Tobramycin	37.86	7	3.22×10^{-6}

Table S4: ANOVA tables of natural logarithm transformed total population density (as OD_{600}) at the final timepoint of the selection experiment. Treatment term refers to the antibiotic treatment regimen, culture refers to the presence or absence of *S. maltophilia*.

Strain	ANOVA term	D.F.	Sum Sq.	F value	P value
PAO1	Treatment	7	1.67	4.42	0.00045
	Culture	1	0.0024	0.044	0.83
	Interaction	7	0.22	0.58	0.77
	Residuals	65	3.50		
LESB58	Treatment	7	2.81	8.30	2.29×10^{-7}
	Culture	1	0.28	5.51	0.022
	Interaction	7	0.53	1.57	0.16
	Residuals	71	3.44		

Supplementary Figures

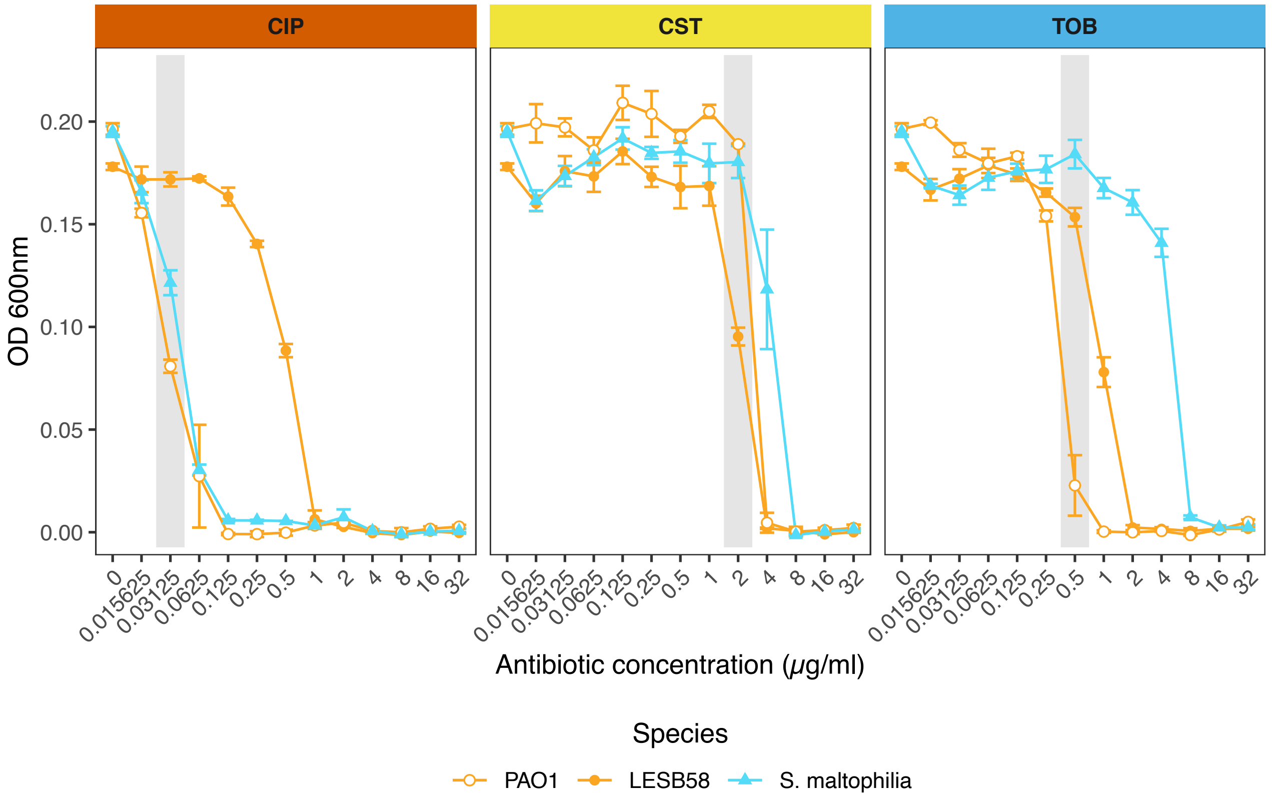

Figure S1: Minimum Inhibitory Concentration curves of ciprofloxacin (CIP), colistin (CST), and tobramycin (TOB) for ancestral *P. aeruginosa* strains PAO1 and LESB58, and *S. maltophilia*.

Highlighted concentration shows the concentration used in the selection experiment. Points show means of triplicate assays, error bars ± SEM.

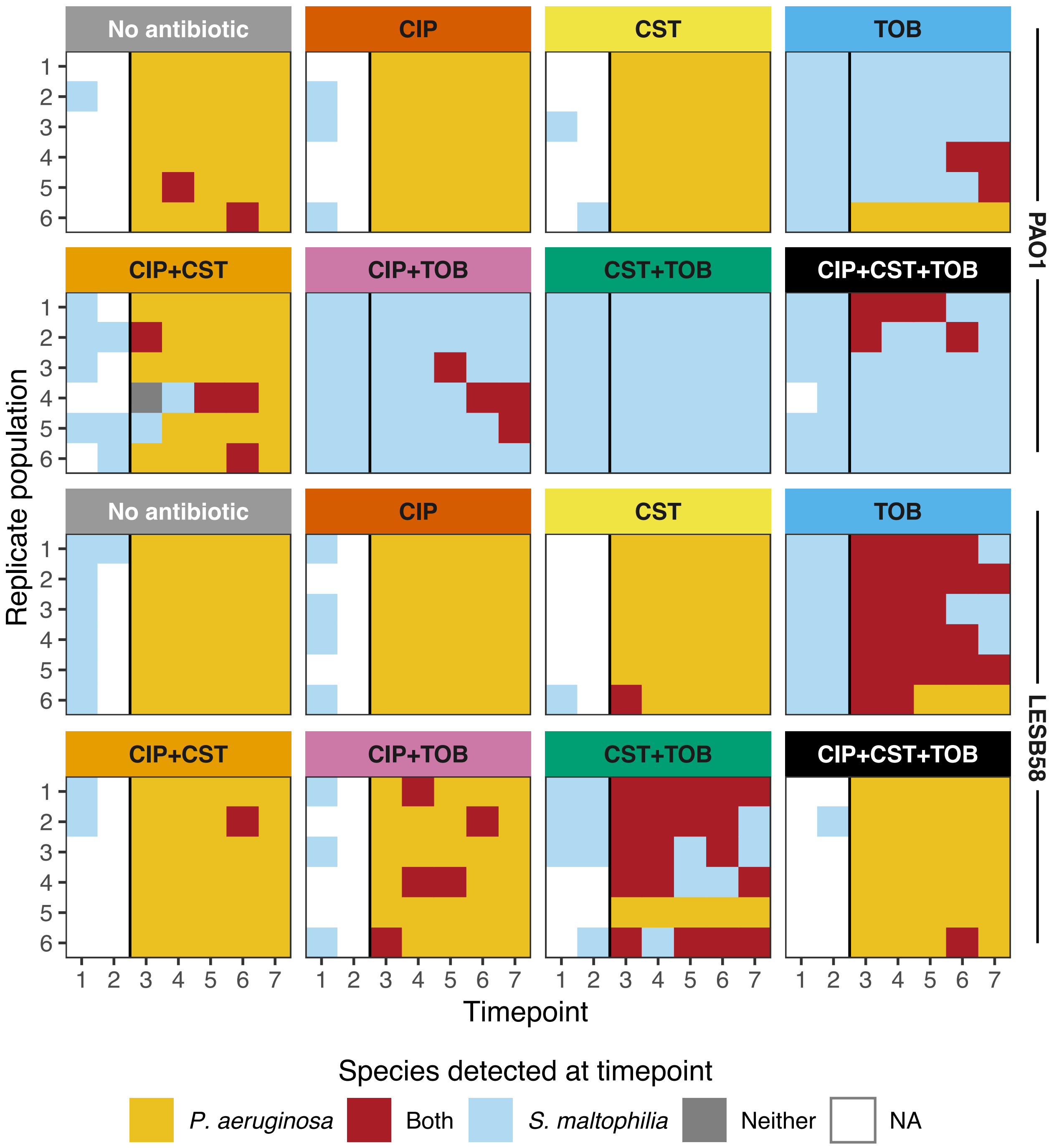

Figure S2: The detected species in each coculture replicate at each timepoint throughout the selection experiment.

Colours represent the surviving species as follows: *P. aeruginosa* in orange, *S. maltophilia* in blue, and both in red; grey represents neither species being detected. *P. aeruginosa* presence in the first two timepoints could not be determined due to failure of the selective agar, shown by white.
